# Selective Autophagy by Close Encounters of the Ubiquitin Kind

**DOI:** 10.3390/cells9112349

**Published:** 2020-10-24

**Authors:** Anna Vainshtein, Paolo Grumati

**Affiliations:** 1 Craft Science Inc., Toronto, ON L4J 7S2, Canada; anna@craftscience.ca; 2 Telethon Institute of Genetics and Medicine, 80078 Pozzuoli (NA), Italy

**Keywords:** selective autophagy, ubiquitin, mitophagy, aggrephagy, lysophagy, xenophagy, lipophagy, nucleophagy, ER-phagy, cargo receptors

## Abstract

Autophagy, a bulk degradation process within eukaryotic cells, is responsible for cellular turnover and nutrient liberation during starvation. Increasing evidence indicate that this process can be extremely discerning. Selective autophagy segregates and eliminates protein aggregates, damaged organelles, and invading organisms. The specificity of this process is largely mediated by post-translational modifications (PTMs), which are recognized by autophagy receptors. These receptors grant autophagy surgical precision in cargo selection, where only tagged substrates are engulfed within autophagosomes and delivered to the lysosome for proteolytic breakdown. A growing number of selective autophagy receptors have emerged including p62, NBR1, OPTN, NDP52, TAX1BP1, TOLLIP, and more continue to be uncovered. The most well-documented PTM is ubiquitination and selective autophagy receptors are equipped with a ubiquitin binding domain and an LC3 interacting region which allows them to physically bridge cargo to autophagosomes. Here, we review the role of ubiquitin and ubiquitin-like post-translational modifications in various types of selective autophagy.

## 1. Introduction

The cellular life cycle is complex, having to contend with ever-changing and at times competing internal and environmental demands, can be stressful. Fail-safe degradation mechanisms are therefore required for the effective disposal of potentially toxic and harmful components and their recycling into building blocks needed for biosynthesis. These degradation systems are vital for the survival and continuity of both long-lived and dividing cells. Several cellular degradation processes have evolved to fill this need and their importance is illustrated through their conservation across evolution, and the pathology that ensues with their perturbance [1,2].

The ubiquitin-proteasome and autophagy-lysosome are the two major cellular degradation systems found in eukaryotic cells and organisms. These processes have remained conserved among species and failure of either one can result in the accumulation of toxic or damaged proteins and organelles, culminating in a number of severe pathologies including cancer, failure to thrive, degenerative diseases, and premature death [1,2]. The ubiquitin-proteasome pathway mainly relies on the 26S proteasome for the final degradation of its substrates [3]. Considered to be the more selective of the two degradation systems, proteasomal substrates are largely composed of individual proteins, requiring large complexes to be disassembled before degradation can take place [4]. The autophagy-lysosome pathway utilizes double membraned vesicles, termed autophagosomes, for the encapsulation and delivery of components to the lysosome for breakdown [5]. Autophagy substrates tend to be larger, including protein aggregates, organelles- either in their entirety or select portions, and invading pathogens. Although autophagy was historically considered to be a bulk degradation pathway, it is now universally accepted that it can be quite selective, with a wide range of substrates under its jurisdiction. Despite the two degradation mechanisms being fairly distinct, they both appear to utilize ubiquitin modification for substrate recognition [6]. It is intriguing that two seemingly independent degradation pathways, which have evolved largely different components and substrates, converge on the same PTM for cargo recognition. This suggests some crosstalk and redundancy between these pathways, with ubiquitin acting as a universal degradation signal.

## 2. Ubiquitin

Ubiquitin (Ub) was originally discovered for its role in tagging proteins for proteasomal degradation. Indeed, Aaron Ciechanover, Avram Hershko, and Irwin Rose received the 2004 Nobel Prize in Chemistry for their discovery and important contributions in unravelling the mechanisms of ubiquitin-mediated proteasomal degradation and the involvement of this pathway in cellular physiology. They demonstrated that ubiquitin was critical for the degradation of proteins involved in cellular regulation, notably cyclins, which play a role in determining cellular replication and mitosis.

Much like its name implies ubiquitin is ubiquitous, it is expressed in all eukaryotic cells and tissues and is evolutionary conserved across species as diverse as plants, yeast, and mammals. A small protein, composed of only 76 amino acids, it is a powerful molecular modifier that governs the fate of proteins and organelles. The ligation of ubiquitin moieties onto a substrate requires a cascade of three distinct enzymes E1, E2, and E3 [3]. E1 is a ubiquitin-activating enzyme that must first prime ubiquitin in an ATP-dependent process. The activated ubiquitin is then transferred to E2, a conjugating enzyme, and finally covalently bonded to a specific substrate with the help of E3, a ubiquitin ligating enzyme. E3 enzymes catalyze the rate-limiting step in Ub conjugation, with over 600 distinct E3 ligases identified so far; this process is extremely substrate-specific and bestows Ub with its selectivity [7]. The ubiquitin tag is then identified by downstream receptors containing a ubiquitin-binding domain (UBD) [8]. Ubiquitin can also be removed from a substrate by deubiquitinating (DUB) enzymes, which antagonize the cell’s ubiquitination machinery [9]. By mediating the degradation of cellular regulators ubiquitin controls a variety of cellular processes including cell cycle, transcription, cellular signaling, metabolism, and more. This molecule is central to cellular decision making and is involved in immune response, development, cell death, and growth [3]. Deficiencies in this process can result in a number of pathologies including cancer and neurodegeneration.

In addition to its central role in proteasomal degradation, ubiquitin has been implicated in autophagy regulation and cargo selection [10]. Substrates can be either monoubiquitinated, through the addition of a single ubiquitin moiety, poly monoubiquitinated through the addition of several single ubiquitin moieties, or polyubiquitinated through the formation of polymeric ubiquitin chains [11]. Although there are multiple possible Ub linkage types, while Lysine 48 (K48) and Lysine 63 (K63)-linked chains are among the most abundant ones [11]. Where K48-linked polyubiquitination more readily targets proteins for proteasomal degradation, K63-linked polyubiquitination appears to be more strongly associated with molecular signaling including autophagy [12]. K63 ubiquitination also regulates protein function, interactions, and localization. Although ubiquitin is itself soluble and remarkably stable, in vitro studies, using uncleavable polyubiquitin moieties, showed that ubiquitin chains, the longer they are, the less stable and prone to aggregation they become [13]. This results in the formation of ubiquitin fibrils, which signal for autophagic clearance. The failure to remove these fibrils results in disease. Evidence suggests that polyubiquitination protects cells from actions by proteins that are destined for degradation, by arresting them in solid aggregates and signaling for their autophagic removal [13]. In this scenario, aberrant autophagy owing to lack of essential autophagy components such as Atg5 or Atg7 results in the accumulation of various types of ubiquitin chain topologies suggesting that autophagy is not dependent on any one specific type of ubiquitination [10].

Thus, one important function of ubiquitin is to deliver the “kiss of death” to proteins, organelles, and invading pathogens. It does so by acting as a molecular beacon for the arrest, aggregation, and removal of substrates by cellular proteolytic systems. For a long time, Ubiquitin was thought to be a lone worrier, however, several functionally and structurally related molecules, collectively termed ubiquitin-like (UBL) proteins have been identified. Among them, LC3s/GABARAPs and ATG12 are the most well-studied in the autophagy field [14,15]. Other UBLs include SUMO, FAT10, ISG15, Hub1/Ubl5, NEDD8, UFM1, and URM1. UBLs share many similarities with Ub, including the three-step conjugation process composed of E1, E2, and E3 enzymes and much like Ub, they are also involved in a growing number of cellular processes, including autophagy [16,17,18,19].

## 3. Autophagy

**Figure 1:**
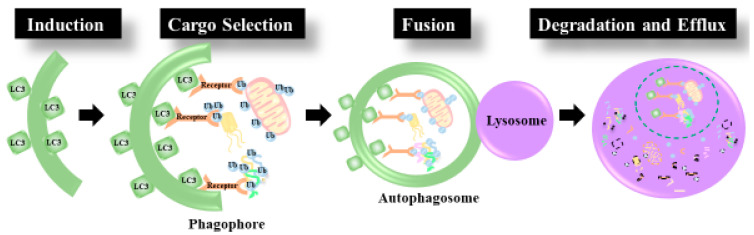
Autophagy Induction: first a pre-autophagosome (phagophore) is formed by the lipidation of LC3. Cargo Selection: Ubiquitinated cargo is tagged for degradation by autophagy receptors equipped with an LC3 binding motif and a ubiquitin binding domain. The Autophagosome is then elongated around the cargo to be degraded. Fusion: the mature autophagosome fuses with the lysosome. Degradation and efflux: The contents of the autophagosome are degraded by proteolytic enzymes within the lysosomal lumen and nutrients are released.

Macroautophagy, herein on termed Autophagy, is a cellular recycling mechanism responsible for cellular housekeeping and turnover during steady-state conditions and nutrient scavenging during starvation. This process involves the formation of double membraned vesicles, known as autophagosomes, around a substrate or a cytoplasmic domain, and the subsequent delivery of these vesicles to the lysosome for proteolytic degradation (Figure 1). Autophagy depends on core autophagy proteins which contribute to the initiation of autophagosome formation, the lipidation of the autophagosomal membrane protein Atg8/LC3/GABARAP, the fusion of the autophagosome to the lysosome, and finally, the degradation of the autophagosome with its cargo within the lysosomal lumen [20]. In 2016 Yoshinori Ohsumi received the Nobel prize in Physiology and Medicine for his discovery of the mechanisms of autophagy in yeast. Ohsumi systematically uncovered autophagy-related genes, classified as ATGs, and his discoveries resulted in an autophagy renaissance.

**Figure 2:**
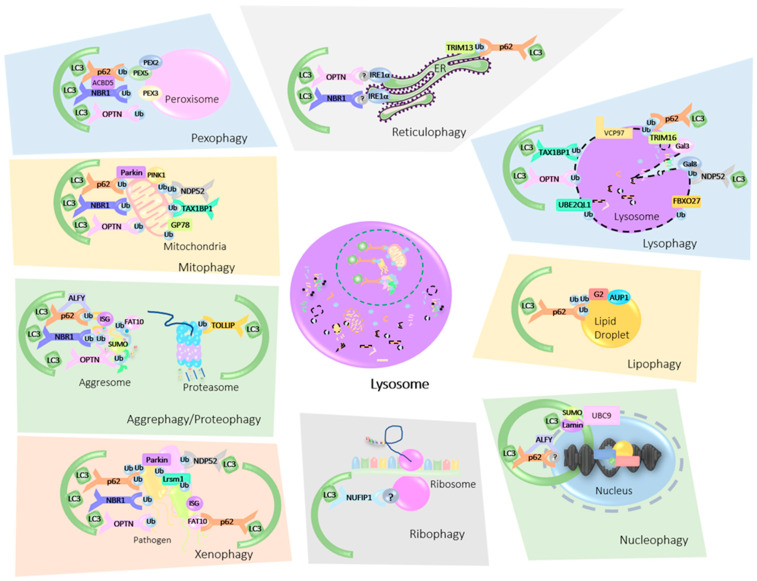
Mammalian Ub-mediated Organellophagy. Damaged, dysfunctional or otherwise superfluous organelles are tagged for degradation by ubiquitin (Ub) or ubiquitin-like molecular modifiers (SUMO, FAT10, ISG15). These post translational modifications (PTMs) are then recognized by autophagy cargo receptors (p62, NBR1, OPTN, NDP52, TAX1BP1, NUFIP1) which bind both the ubiquitinated cargo and the microtubule-associated protein 1B light chain 3B (LC3B). The autophagosome engulfs the receptor cargo complex and delivers it to the lysosome for breakdown. *ER-Phagy/Reticulophagy***:** ER stress sensor IRE1α, is activated upon ER stress. IRE1α interacts with p62, OPTN, and NBR1 to promote reticulophagy, but whether Ub is involved in this process is unclear. Self-ubiquitination of the E3 ligase TRIM13 also recruits p62 to damaged ER. This results in the sequestration of the ER within autophagosomes and its delivery to the lysosome for elimination. *Pexophagy***:** PEX2 ubiquitinates the peroxisomal import receptor PEX5 which is then recognized by autophagy receptors p62 and NBR1. PEX3 is also involved in this process and enhances peroxisomal ubiquitination, while peroxisomal acyl-CoA binding domain containing 5 (ACBD5) regulates the pexophagy receptor protein complex. OPTN was also shown to be involved in this process. *Mitophagy*: Mitochondrial damage results in the stabilization of PINK1 on the mitochondrial outer membrane, which entices the arrival of E3 ligases Parkin and GP78. These ligases, in turn, ubiquitinate a variety of substrates on the mitochondrial membrane which recruits autophagy receptors p62, NDP52, OPTN, NBR1, and TAX1BP1. *Aggrephagy/Proteophagy:* Protein aggregates are recognized by selective autophagy receptors p62, NBR1, OPTN, and TOLLIP, with autophagy-linked FYVE protein (ALFY) acting as a scaffold protein promoting the assembly of p62 bodies. Cue5 the yeast homolog of mammalian TOLLIP binds ubiquitinated proteasomes targeting them for proteophagy. Ubiquitin and ubiquitin like PTMs (UBLs SUMO, FAT10, and ISG15) also play a role in aggrephagy. *Xenophagy:* The E3 ligases Parkin and Lrsm1 ubiquitinate invading pathogens which are then detected by p62, NBR1, NDP52, and OPTN. Other UBLs have also been demonstrated to promote xenophagy including FAT10 and ISG15. With FAT10 interacting directly with p62. Recognition by cargo receptors then promotes the incarceration of pathogens within autophagosomes and their delivery to lysosomes for elimination. *Ribophagy:* The large ribosomal subunit is targeted for autophagosomal degradation by the cargo receptor NUFIP1, however, the involvement of Ub or other UBLs in this process has not been determined. *Nucleophagy:* The selective autophagy receptor p62 and autophagic adaptor ALFY, have been documented to shuttle between the nucleus and cytoplasm, however whether Ub is involved in this process is not known. SUMOylation, was documented to drive nucleophagy where DNA damage promotes the accumulation of SUMO E2 ligase UBC9, resulting in the SUMOylation of lamin A/C. *Lipophagy:* Ancient Ubiquitous Protein 1 (AUP1) localizes to LDs and recruits the E2 ubiquitin-conjugating enzyme G2, which ubiquitinates targets on the lipid droplet, thus inviting autophagy receptor p62. *Lysophagy:* Glycans are exposed following lysosomal membrane rapture and are identified by galectins-3 and -8 (Gal3/8) or by the SCF ubiquitin ligase FBXO27. Gal3 then stimulates ubiquitination by TRIM16 and recruits the autophagy receptor p62, while Gal8 engages NDP52. The E2 ubiquitin-conjugating enzyme UBE2QL1 was also found to participate in this process, as were autophagy receptors TAX1BP1 and OPTN. The AAA-ATPase VCP97 is another essential player in the elimination of injured lysosomes.

Once considered an indiscriminate bulk degradation process, autophagy is now well recognized for its selectivity. With the aid of various receptors and signaling molecules, autophagy can discern between healthy cellular components and toxic organelles, proteins, as well as invading organisms. Indeed, constitutive autophagy can be extremely selective and is responsible for the removal of cellular components, like misfolded protein aggregates or exhausted organelles, to ensure a proper physiological turnover [21]. Moreover, autophagy induced by a specific trigger can also be very selective; for instance, xenophagy induced by a pathogen infection triggers a selective autophagic response against the invading organism. Likewise, when mitochondria are chemically depolarized, mitophagy ensues and targets strictly depolarized mitochondria [22,23,24]. Contrarily, autophagy induced by severe nutrient depletion may not be overly selective, as its sole goal is nutrient liberation under conditions of cellular stress. With this in mind, various types of substrates are eliminated through selective autophagy and this number is continuously on the rise. These include protein aggregates (aggrephagy/proteophagy), mitochondria (mitophagy), endoplasmic reticulum (reticulophagy/ER-phagy), peroxisome (pexophagy), nucleus (nucleophagy), pathogens (xenophagy), lipids (lipophagy), and even lysosomes themselves (lysophagy) (Figure 2) [23,25]. During selective autophagy, dysfunctional or obsolete proteins and organelles are targeted for degradation by various receptors, which, in turn, entice the arrival of the autophagosome. The selectivity in this process is largely mediated by PTMs such as phosphorylation and ubiquitination, with UFMylation, ISGylation, and SUMOylation recently arising as potential PTMs, similar to Ubiquitination, that modulate autophagy [17].

Moreover, there are two ubiquitin-like (UBL) protein conjugation systems that act to promote the formation of the autophagosome precursor, the phagophore [26]. These are involved in the conjugation of Atg12, and ultimately, microtubule-associated protein light chain 3 (LC3) and its close relatives GABARAP and GATE16 [27,28,29]. One pathway involves the covalent conjugation of Atg12 to Atg5. This occurs with the help of Atg7 and Atg10, E1- and E2-like enzymes, respectively. The Atg12–Atg5 heteromer then interacts with Atg16-like 1 (Atg16L1) [30]. This complex acts as a *de facto* E3-like ligase to promote the second conjugation reaction, involving LC3. The conjugation of LC3 to phosphatidylethanolamine (PE) is essential for phagophore expansion and sealing. LC3 exists in its inactive form free in the cytosol and must first be cleaved by the protease Atg4 giving rise to LC3-I [31]. The E1-like enzyme Atg7 and the E2-like enzyme Atg3 are then tasked with priming LC3-I for its final conjugation to PE, by the E3-like complex of Atg12-Atg5. Lipid conjugation converts LC3-I, to the LC3-II form that is attached to either side of the growing phagophore membrane.

## 4. Selective Autophagy

The benefits of selective autophagy are vast, as it allows for the surgical removal of targeted substrates. For instance, the removal of mitochondria during hypoxia, organelle damage, or even when the organelle becomes obsolete, such as during erythrocyte maturation, all occur selectively through mitophagy [32,33,34]. In bulk autophagy, the ubiquitination of autophagic components often acts as a regulatory signal. However, when it comes to selective autophagy ubiquitination also acts as a signal for cargo recognition, process initiation, and rate of autophagosome formation. For instance, the ubiquitination of components of the PI3K-Beclin1 complex contributes to the regulation of autophagic induction and autophagosome formation [35,36]. Selective autophagy relies on core autophagy components but is also aided by specific selective autophagy receptors. The cargo selection step of this process is highly dependent on these receptors (Table 1).

Generally, during selective autophagy, protein aggregates, damaged organelles or portions thereof are identified and tagged for degradation by E3 Ub-ligases. Once cargos are tagged, ubiquitin acts as an “eat me” signal for autophagy receptors, which further flag targets for degradation. Autophagy receptors are also implicated in segregating and coalescing materials destined for autophagic degradation, effectively preparing them for the arrival of the phagophore. Indeed, autophagy receptors harbor a UBL domain to sense Ub molecules, and a LIR domain to bind the LC3s/GABARAPs, which are present on autophagosomal membranes. By binding to mATG8s, cargo receptors promote the recruitment of autophagosomal membranes around the Ub-tagged materials, eventually encapsulating them completely. Mature autophagosomes are then delivered to the lysosome for degradation [37,38].

Considering that potentially all cytosolic components could be selectively degraded via lysosomes, it is plausible that selective autophagy plays a role in a number of diverse physiological processes and their associated diseases. The ability to specifically target the elimination of desired cellular constituents presents great therapeutic potential for the treatment of many diseases characterized by the accumulation of toxic materials including neurodegenerative diseases like Huntington’s and Alzheimer’s, lysosomal storage diseases, and mitochondrial DNA disorders [39,40]. Moreover, selective autophagy has also been implicated in chronic obstructive pulmonary disease (COPD) [41] as well as other pulmonary disorders [42]. Of note, one of the most well-studied functions of selective autophagy in human health is its role in infections [43]. Once bacteria invade the host cell, they are immediately labeled with ubiquitin chains and selectively degraded via xenophagy [44]. The removal of viral pathogens by selective autophagy is also termed virophagy [45,46]. Of note, host cells counteract some types of viral infections, like flaviviruses and Ebola virus, by directly eliminating their replicative niche in the endoplasmic reticulum, thus activating another form of selective autophagy named ER-phagy [47,48].

## 5. Cargo Receptors

Most autophagy cargo receptors are characterized by the presence of a ubiquitin-binding domain (UBD), enabling the detection of marked cargo, and an LC3-interacting region (LIR) allowing for interaction with LC3/GABARAP/GATE16 proteins on the autophagosomal membrane [8]. These properties permit receptors to act as middlemen, effectively bridging tagged cargos with autophagosomes. Indeed, receptors accompany their targets to the lysosome and are degraded alongside them. The UBD, composed of either a UBA, UBAN (ubiquitin-binding in ABIN and NEMO), or ubiquitin-binding zinc finger motif, can recognize various flavors of Ub chain linkages, and its affinity for ubiquitinated substrates can be further enhanced through phosphorylation [49,50,51]. The core of the LIR motif consists of a minimal consensus sequence W/F/Y-x-x-L/V/I, which is common to most cargo receptors [52], and the phosphorylation of this domain was also demonstrated to augment binding affinity to LC3 and related proteins [53]. Of note, some cargo receptors like NDP52 and TAX1BP1 present an unconventional LIR domain (LVV) that is missing the consensus aromatic amino acid. This non-canonical LIR is important for the function of NDP52 and TAX1BP1 in xenophagy and seems to confer the receptor preferential binding to LC3C, at least in the case of NDP52 [54,55].

Although LIR was the first domain recognized to mediate the binding of ATG8s to autophagy adaptors and receptors, another domain with a similar function has been recently characterized. Several ATG8 interactors exploit a ubiquitin-interacting motif (UIM)-like sequence that confers them with a high affinity for autophagy modifiers [56]. However, the presence of a LIR or UIM motif does not “a priori” confer cargo receptors ATG8-binding abilities. A real interaction should always be experimentally confirmed. Nevertheless, the characterization of the UIM domain greatly expands the number of cellular proteins that could serve as autophagy receptors and adaptors.

The first cargo receptor to be identified was p62 and it is undoubtedly the most versatile and well-studied receptor to date. p62 is ubiquitously expressed and mediates the removal of a wide range of ubiquitinated proteins, protein aggregates, and organelles [57]. Due to its common role in sequestering ubiquitin positive protein aggregates, it earned the name sequestosome-1 (SQSTM1) and was demonstrated to selectively bind to ubiquitinated protein aggregates such as neurofibrillary tangles, Lewy bodies, tau and α-synuclein and alike [58]. p62 contains both a UBA and an LIR domain, thus mediating the association between cargos and autophagosomes [59]. p62 itself is degraded alongside its cargo, as it accompanies the targeted components to their final destination. Indeed, conditions that are characterized by defective autophagy often display large accumulations of p62 along with ubiquitinated proteins. Interestingly, in some cases elimination of p62 in autophagy-deficient models results in protein aggregate clearance [10], suggesting a role for p62 in the coalescing of materials for autophagic degradation. In addition to p62, many other cargo receptors have been described to participate in various types of selective autophagy including NBR1 (neighbor of BRCA1 gene 1), NDP52 (nuclear dot protein 52 kDa), Optineurin (OPTN), TAX1BP1 (Tax1-binding protein 1), and TOLLIP (Toll-interacting protein) (Table 1), with the number of receptors continuing to grow as more types of selective autophagy are identified.

A screen for Atg8 interactors identified NBR1 as an autophagy receptor containing both LIR and a UBA ubiquitin-binding domain. NBR1 is recruited to Ub-positive protein aggregates and is degraded by autophagy, a process that requires a functional LIR domain [49,60,61,62]. NBR1 and p62 both act as selective autophagy receptors but appear to function independently as NBR1-positive aggregates are cleared by autophagosomes even in the absence of p62. The depletion of NBR1 abolishes the formation of Ub-positive p62 bodies upon the puromycin treatment of cells [61].

Similar to p62 and NBR1, Optineurin (OPTN) is a selective autophagy receptor equipped with a UBAN-type UBD and a LIR [50,53]. OPTN is present in protein inclusions observed in various neurodegenerative diseases including amyotrophic lateral sclerosis (ALS), Huntington’s, Alzheimer’s, Parkinson’s, Creutzfeld-Jacob’s, and Pick’s disease. Its depletion significantly increases protein aggregation in HeLa cells and morpholino-silencing of the OPTN ortholog in zebrafish causes a motor axonopathy phenotype similar to ALS [63]. Mutations in OPTN’s UBAN and LIR domains abolish its ability to bind Ub and LC3/GABARAP modifiers, respectively; these mutations do not appear to compromise its ability to colocalize with protein aggregates, suggesting that OPTN interacts with protein aggregates in a manner that is ubiquitin and LC3 -independent [53]. Indeed, OPTN recognizes various protein aggregates via its C-terminal coiled-coil domain in a ubiquitin-independent manner. Moreover, TANK1 binding kinase 1 (TBK1) phosphorylates OPTN, which regulates its ability to interact with autophagy modifiers and clear protein aggregates [53,63]. OPTN can itself be ubiquitinated by the E3 ligase HACE1 (HECT domain and ankyrin repeat-containing E3 Ub protein ligase 1) which promotes its complexing with p62 and enhances autophagy flux [64]. The HACE1-OPTN interaction synergistically suppresses the growth and tumorigenicity of lung cancer cells [64].

Thus, selective autophagy relies on receptors for the targeting and coalescing of materials into autophagosomes for degradation. Numerous such receptors have been identified, each with its own repertoire of autophagy targets.

## 6. Mitophagy

Mitochondria, the powerhouse of the cell, provide energy in the form of ATP to fuel most cellular activities. However, these powerful organelles also produce toxic reactive oxygen species (ROS) as a by-product of oxidative phosphorylation. This leaves them vulnerable to ROS-induced damage and dysfunction. Recycling damaged mitochondria is therefore vital for cellular health and bioenergetics. The wholesale removal of mitochondria occurs through a selective process termed mitophagy. Mitophagy is ever-ongoing at low levels and accounts for routine mitochondrial turnover which occurs approximately every 7–14 days, but may be accelerated during energetic imbalance or oxidative stress [65]. Mitophagy also participates in the pruning of healthy mitochondria during various physiological processes such as erythrocyte maturation and the elimination of paternal mitochondria during oocyte fertilization [34,66,67,68]. Interestingly, the stimulation of mitochondrial oxidative phosphorylation has also been demonstrated to enhance mitophagy through the small GTPase Rheb [69]. This acts as a pre-emptive mechanism to facilitate mitochondrial renewal in response to enhanced metabolic demands. Here, we provide evidence to the role of ubiquitin in targeting mitochondria for autophagic degradation, mechanisms of mitophagy have been extensively reviewed elsewhere and are beyond the scope of this review [22,24].

When mitochondria withstand irreversible damage and all other quality control mechanisms have failed to restore proper mitochondrial function, mitophagy is triggered. The loss of mitochondrial membrane potential and integrity results in the stabilization of mitochondrial health sensor PINK1 (PTEN-induced putative kinase protein 1) on the organelle’s outer membrane. PINK1 serves as a docking station for the arrival of the cytosolic E3 ubiquitin ligase Parkin to damaged mitochondria. The phosphorylation of Parkin and ubiquitin chains by PINK1 ensures Parkin is retained on the surface of depolarized mitochondria effectively tagging damaged mitochondria for degradation [70,71,72,73]. To keep mitochondria stationary, PINK1 phosphorylates Miro, a component of the motor adaptor complex. Miro is responsible for anchoring the motor protein kinesin to the mitochondrial surface and facilitating mitochondrial movement along microtubule tracks. Miro phosphorylation predisposes it for degradation in a Parkin-dependent manner [74] thus, keeping mitochondria at a standstill. Moreover, Parkin also mediates the ubiquitination and degradation of mitochondrial fusion effectors, Mitofusins 1 and 2 (MFN1 and MFN2). This ensures the isolation of the damaged organelle. Parkin has also been documented to ubiquitinate a variety of other outer mitochondrial membrane proteins such as the voltage-dependent anion channels (VDACs), translocases of the outer membrane (TOMs), as well as many others [73]. The ER-associated E3 ligase Glycoprotein 78 (Gp78) was also found to ubiquitinate mitochondrial outer membrane proteins such as MFN1 and induce mitophagy of depolarized mitochondria independently of Parkin [75]. On the other hand, deubiquitinase USP30 (ubiquitin carboxyl-terminal hydrolase 30) acts in opposition to Parkin, removing Ub from depolarized mitochondria thus blocking mitophagy [76]. Defective mitophagy, resulting from deficiencies in Parkin or PINK1 can be rescued by the knockdown of USP30 [76]. The presence of ubiquitinated proteins on the mitochondrial outer membrane, act as signals for the arrival of autophagy receptors NBR1, p62, OPTN, NDP52, and TAX1BP1 [61,77,78,79]. However, some evidence suggests that p62 may not be required for mitochondrial degradation by autophagy [22,79,80]. Indeed, a comprehensive study demonstrated NDP52 and OPTN to be the main receptors responsible for PINK1- and Parkin-mediated mitophagy, where they also activate and recruit core autophagic machinery [79].

## 7. Aggrephagy/Proteophagy

Busy making proteins to sustain life, cells make mistakes in protein folding, proteins get damaged and oxidized by toxic metabolic byproducts, and certain proteins, such as polyglutamine (polyQ), are inherently prone to aggregation. Aggrephagy refers to the autophagic degradation of protein aggregates, which aberrantly accumulate due to processing mistakes, damage, or proteasomal dysfunction [81]. Protein aggregates are common in proteinopathies such as neurodegenerative diseases and aging [21]. The usual aggregate prone suspects, including amyloid-β [82], huntingtin [83,84], and alpha synuclein [85], can all be degraded through this process. Ubiquitination is vital for the autophagic removal of protein aggregates in proteinopathies [86,87,88,89], with p62, NBR1, and OPTN all participating as cargo receptors in mammals [23]. Scaffolding proteins such as ALFY also appear to play a role in this process by promoting the assembly of p62 bodies and their degradation by autophagy [90,91]. The most recently identified aggrephagy receptor, in yeast, is Cue5 (coupling of ubiquitin to ER degradation-5). Cue5 promotes aggrephagy by facilitating the interaction between Ub and Atg8 on protein aggregates. Cue5 also acts as an autophagy receptor for proteasomal subunit degradation, known as proteophagy, by binding to ubiquitinated proteasomes and promoting their removal by autophagy [92,93,94]. The overexpression of TOLLIP, the human homolog of Cue5 has been demonstrated to mediate the degradation of polyQ proteins in human cell lines [95].

Moreover, other UBLs including SUMOylation (the post-translational modification of proteins with small ubiquitin-like modifiers), FAT10 (the addition of the cytokine-inducible HLA-F adjacent transcript 10, also known as ubiquitin D, onto proteins) and ISGylation (the covalent attachment of Interferon-stimulated gene 15 (ISG15) to substrates) have been documented to play a role in aggrephagy. SUMOylation, like ubiquitination, involves lysine residue modifications of proteins through a three-step process involving distinct enzymes: SUMO E1 (the heterodimer SAE1 and SAE2), SUMO E2-conjugating enzyme (UBE2I/UBC9) and several E3 ligases [21,22]. SUMO-1 was found to accelerate the accumulation of autophagic vacuoles in neurons, thus increasing the production of amyloid-β (Aβ) [96]. FAT10 was found to regulate the solubility of polyQ proteins, such as huntingtin [97], and ensure the delivery of aggregated or damaged proteins to the aggresome during proteasome dysfunction by interacting with histone deacetylase 6 (HDAC6) [98]. Similarly, ISGylation marks protein aggregates for autophagic disposal and also interacts with p62 and HDAC6 to enhance aggrephagy [99].

## 8. ER-Phagy/Reticulophagy

The endoplasmic reticulum is a jack of many trades, involved in proper protein folding, processing and secretion, as well as calcium homeostasis, and lipid synthesis. The ER has also been documented to contribute membranes to growing autophagosomes [100,101,102]. The ER is a continuous membrane structure composed of an extensive network of sheets and tubules [103]. The accumulation of unfolded proteins induces an ER stress response known as the unfolded protein response (UPR). This program activates the following ER stress sensors: inositol-requiring protein 1 (IRE1), protein kinase RNA-like ER kinase (PERK) and activating transcription factor 6 (ATF6) [104], which bring protein synthesis to a halt, enhance the degradation of misfolded proteins, and increase the expression of molecular chaperones. If these actions do not resolve ER stress, the selective autophagy of the ER or ER-phagy will ensue in a final attempt to avoid the induction of apoptosis [105,106]. Through this process, the ER undergoes significant remodeling and is targeted for degradation by the following cargo receptors: FAM134B, SEC62, RTN3, CCPG1, ATL3, and TEX264 [107,108,109,110,111,112,113]. Thus far, the role of ubiquitin in ER-phagy, and its relationship with cargo receptors involved in this process, remains largely unknown. However, some recent data suggest that ubiquitination does indeed play a role in this process. In this scenario, the N-degron pathway mediates ER-phagy through the auto-ubiquitination of the transmembrane E3 ligase TRIM13. Once ubiquitinated, TRIM13 signals for the arrival of cargo receptor p62 thus recruiting the phagophore to the damaged organelle. This is dependent on an N-degron destabilizing residue on the N-terminal arginine (Nt-Arg) [114]. p62, in turn, facilitates the encapsulation of ER into autophagosomes and their turnover in the lysosome, by binding to ER transmembrane protein IRE1α [115,116]. IRE1α has also been demonstrated to bind other Ub binding receptors such as OPTN and NBR1 [115].

## 9. Ribophagy

Ribosomes are the cellular protein manufacturing facilities and can be found free-floating in the cytosol or attached to the ER. These tiny factories sustain life through continuous protein biosynthesis. During starvation, ribosomes are selectively eliminated via ribophagy to generate nucleotides/nucleosides required for cellular survival [117].

In yeast selective degradation of mature ribosomes is mediated by the Ubp3p ubiquitin protease complex composed of Ubp3p, Bre5, Cdc48, and Ufd3 [117,118]. This complex deubiquitinates Rpl25 on the large ribosomal subunit, which results in its degradation. This process is antagonized by Ltn1-mediated ubiquitination of the same residue on Rpl25, creating a dynamic and selective ribophagy signal. Therefore, ubiquitin-mediated regulation of ribophagy, in yeast, is counterintuitive to other types of selective autophagy. Where, in most other types of selective autophagy ubiquitination functions as a marker for cargo recognition, in ribophagy ubiquitination appears to be protective, and must be removed to trigger autophagic degradation, at least in yeast. Interestingly this interplay only controls the turnover of the large ribosomal subunit. This suggests ribophagy is even more selective as independent mechanisms are responsible for the degradation of the different ribosomal subunits [117].

Ribophagy has also been demonstrated to occur in human cells in response to starvation, mTOR inhibition, and treatment with cellular stressors such as sodium arsenite and reversine [119]. However, the molecular mechanisms that regulate ribophagy are not clear and some controversial theories co-exist. NUFIP1 (nuclear fragile X mental retardation–interacting protein 1) was initially characterized as a selective ribophagy receptor, responsible for mediating ribosomal degradation by directly interacting with LC3B and ribosomes during starvation [120]. Moreover, ribophagy appeared to be particularly important during nutrient depletion as it represents a source of nucleosides for starving cells [120]. Further studies on ribophagy revealed that the delivery of ribosomes to lysosomes could follow one of three paths: (i) random engulfment of ribosomes into autophagosomes during bulk autophagy; (ii) by-stander flux following ER-phagy induction and (iii) receptor-mediated selective autophagy [113,119]. Moreover, recent proteomic studies revealed that during acute nutrient stress, ribosomal protein degradation occurs mainly via a non-autophagic pathway. No obvious differences in ribosomal protein levels were detected between wild-type and *ATG7* or *RB1CC1* deficient cells following treatment with Torin 1. Moreover, similar results were obtained in NUFIP1 deficient cells, bringing the role of this protein in ribophagy into question [121].

Although the involvement of ubiquitination in ribophagy has not been determined yet, ribosomal protein ubiquitination has been demonstrated to occur under stressful conditions such as translation stalling, oxidative stress, and UPR [122,123,124,125]. Indeed, the E3 ligases HUWE1 (HECT, UBA, and WWE Domain Containing Protein 1) and UBE2O (Ubiquitin Conjugating Enzyme E2 O) have been implicated in the ubiquitination and proteasomal degradation of ribosomal proteins [126,127].

## 10. Pexophagy

Peroxisomes are small organelles responsible for lipid metabolism and redox regulation in most eukaryotic cells. As both producers and scavengers of reactive oxygen species (ROS), these organelles play a multipurpose role in cellular signaling and metabolism. The peroxisomal lifecycle is quite dynamic, with a half-life of only about two days. The elimination of peroxisomes takes place through a selective autophagy process termed pexophagy. Like other selective autophagy substrates, peroxisomes must be ubiquitinated and decorated by cargo receptors to be targeted for elimination.

PEX5 is an import receptor that shuttles between the peroxisomal membrane and the cytosol. Phosphorylation of PEX5 by ataxia-telangiectasia mutated (ATM) predisposes it to mono-ubiquitination, which blocks its export and signals for peroxisomal degradation by autophagy [128,129]. The E3 ubiquitin ligase Peroxin 2 (PEX2) has been implicated in the ubiquitination of PEX5, and PMP70 during starvation [130]. Moreover, PEX14, a peroxisomal membrane protein that acts as PEX5′s docking partner can directly interact with LC3B-II during starvation. An additional peroxisomal import factor, PEX3, has also been documented to play a role in pexophagy, where the overexpression of PEX3 induces ubiquitination and elimination of peroxisomes by autophagy [131].

Selective autophagy receptors NBR1 [62] and p62 [132] were both shown to participate in this process. Indeed, NBR1 was demonstrated to be both sufficient and required to drive peroxisomal elimination by pexophagy, a role that requires its amphipathic α-helical J domain, ubiquitin-associated (UBA) domain, LC3-interacting region, and coiled-coil domain. p62 binding to NBR1 does not appear required for its function but does enhance pexophagy [62]. Moreover, some emerging evidence is suggesting that the peroxisomal protein ACBD5 (Acyl-CoA-binding domain containing protein 5), may be important for regulating the pexophagy receptor protein complex [133]. However, pexophagy research is still in its infancy and more studies are required to further elucidate the molecular mechanisms regulating this process.

## 11. Lysophagy

Lysosomes are acidic membrane-bound organelles, which contain a wide variety of hydrolytic enzymes required for the degradation of various substrates including proteins, lipids, carbohydrates, nucleic acids, and invading pathogens. Lysosomes play a key role in cellular homeostasis by controlling both cellular clearance, and energy production in response to environmental cues [134]. These organelles contain a slew of hydrolases (e.g., proteases, nucleases, esterases, polysaccharidases, and glycosidases) capable of degrading a wide spectrum of components. Therefore, upon injury the spilling of lysosomal contents poses a threat to cellular health and can culminate in inflammation and cell death [135,136]. It is not surprising then that damaged lysosomes themselves undergo a selective form of autophagy, termed lysophagy.

Several cellular events can render the lysosomal membrane prone to permeabilization including excessive cholesterol, toxic aggregates such as α-synuclein, mutant huntingtin, and Aβ or tau fibrils, as well as alterations in lipid composition and ROS, just to name a few. Physical damage to lysosomes induced by silica, lysosomotropic agents, and urate crystals have all been documented to induce lysophagy [137]. Upon lysosomal membrane rupture and failing any repair efforts, glycans that normally reside within the lysosomal lumen are exposed. These glycans are then identified by galectins-3 and -8 or by the SCF ubiquitin ligase, FBXO27, leading to the ubiquitination of lysosomal components [138,139]. Indeed, Galectin-3 (Gal3) stimulates the ubiquitination of lysosomal proteins by TRIM16 [140,141], thus recruiting the autophagy receptor p62, while Gal8 engages NDP52 [142].

Moreover, it appears that both K63- and K48-linked ubiquitination are involved in lysophagy. The E2 ubiquitin-conjugating enzyme UBE2QL1 ubiquitinates lysosomal membrane proteins with K48-linked Ub chains [4,143]. While p97 (Valosin-containing protein) translocates to the lysosome to form a complex with UBXD1, PLAA, and YOD1. This complex acts downstream of K63 ubiquitin chains and removes K48-linked ubiquitin conjugates from damaged lysosomes to promote the recruitment of autophagic membranes [4].

In this process ubiquitinated lysosomes recruit autophagy receptors which promote their engulfment within autophagosomes. They are subsequently cannibalized by healthy lysosomes [144]. Interestingly, multiple cargo receptors have been demonstrated to play a role in this process including p62 [4,145], NDP52 [142], TAX1BP1 [143], and OPTN [146]. These are essential for forming a docking platform for ULK1, which activates the core autophagic machinery and induces autophagosome formation regulated by TBK1 [146].

This fits well within the autophagy wheelhouse and can be particularly useful for host-defense. Indeed, one-way pathogens are able to establish an infection, is by breaking vacuolar membranes and escaping into the cytoplasm [147]. This has been documented during Shigella infection, where host cell-membrane remnants attached to a bacterium, activate an autophagic response resulting in pathogen removal [148].

## 12. Xenophagy

Xenophagy refers to the selective removal of invading pathogens and constitutes an important faction of innate and acquired immune responses. Autophagy plays a role in combating a number of pathogens including bacteria such as *Streptococcus pyogenes*, *Mycobacterium tuberculosis*, *Listeria monocytogenes, Shigella flexneri, Salmonella enterica,* and *Toxoplasma gondii.* Similarly, viruses such as Sindbis are also targeted by autophagy [149]. Like other forms of selective autophagy, pathogens are first tagged for degradation by Ub [148,150,151,152] and are then recognized by cargo receptors. This process also requires functional core autophagy machinery. Ubiquitin is vital for xenophagy and is responsible for linking bacteria-containing endosomes with autophagic machinery, such as Atg16L1 [145]. Some examples of E3 ubiquitin ligases that function as xenophagy regulators are: ARIH1/HHARI (ariadne homologue 1) and LRSAM1 (leucine rich repeat and sterile alpha motif containing 1) that ubiquitinate cytosolic *Salmonella Typhimurium* [153,154]. Linear ubiquitin chains have also been detected on the surface of Salmonella; therefore, the E3 complex LUBAC (linear ubiquitin chain assembly complex) and its specific DUB OTULIN (OTU Deubiquitinase With Linear Linkage Specificity) play an active role in xenophagy [155,156]. Moreover, Parkin was shown to ubiquitinate invading bacteria *Mycobacterium tuberculosis* [157]. The cargo receptors p62 [158,159], NBR1 [61], NDP52 [158,160], and OPTN [53] have all been demonstrated to facilitate the sequestration of invading bacteria in autophagic vesicles.

Interestingly, other PTMs such as interferon-α,β inducible ubiquitin-like modifiers FAT10 and ISG15 have also been implicated in xenophagy. Where FAT10 was found to decorate autophagy-targeted *Salmonella* and recruit p62, thus contributing to resistance against *Salmonella* infection in mice, through the induction of xenophagy [18]. Similarly, ISG15 was found to be essential for optimal mycobacterial immunity, by mediating the release of IFN-γ-inducing secreted molecule [161,162]. Indeed, enhanced ISGylation in cells and animals results in greater basal and infection-induced autophagy. This is largely mediated by temporary metabolic reprogramming induced by the ISGylation of mTOR, WIPI2, and AMBRA1 following infection [16].

## 13. Lipophagy

Autophagic degradation of lipid droplets, the organelles responsible for cellular lipid storage, is termed lipophagy. Although it was first described over a decade ago, the molecular mechanisms involved in this process remain largely elusive [163]. Lipophagy is particularly important in the liver, where it is responsible for lipid mobilization in response to a multitude of cellular stressors. Indeed, lipid droplets were found to co-localize with autophagy marker LC3B in mouse liver [163]. Later studies uncovered that Huntingtin, the protein mutated in Huntington’s disease, is involved in stress-induced lipophagy and appears to act by facilitating the binding between LC3B and p62 [164]. Another protein, Ancient Ubiquitous Protein 1 (AUP1), has been shown to localize to LDs and act as a recruiter for the E2 ubiquitin conjugase G2 (Ube2g2). These findings were the first to suggest that the ubiquitination machinery localizes to LDs and promotes their degradation by autophagy [165]. Moreover, ethanol-induced lipophagy occurs through the increased presence of Ub and p62 on LDs [166]. It appears that ubiquitin may play a role in this selective autophagy process, but solid evidence to this effect, as well as the molecular mechanisms involved, remain to be illuminated.

## 14. Nucleophagy

The nucleus is the “brain” of the cell, entrusted with safeguarding the genome and dictating cellular activities by controlling gene expression. The health and integrity of this organelle is therefore the cell’s priority. Nuclear recycling by autophagy, known as nucleophagy, is an evolutionarily conserved process among eukaryotes and is responsible for the degradation of nuclear components. Although in some multinucleated eukaryotic cells the nucleus is degraded wholesale by autophagy [167], in most eukaryotes, nucleophagy is thought to occur piecemeal, and require a series of events [168]. This process begins by first sensing and flagging damaged components which are then exported from the nucleus, encapsulated within the autophagosome, and eventually degraded by the lysosome. The precise molecular mechanisms, and whether protein ubiquitination is involved, remain largely unexplored.

In yeast nuclear recycling was documented to occur piecemeal through micronucleophagy [169,170], or by macronucleophagy with the help of the autophagy receptor Atg39 [171], under physiological and nutrient stress conditions, respectively. Nucleophagy has also been described to occur in differentiating murine and human keratinocyte, where perinuclear vesicles containing histone interacting protein and heterochromatin protein 1α, were shown to localize near Lamin A and B1 [172]. Although nucleophagy is not well understood in mammals, dysfunction in this process appears to contribute to pathologies such as cancer and neurodegeneration and is also implicated in cellular senescence and aging [168]. It is unclear, however, whether nucleophagy is an ongoing physiological process that is further triggered by pathology, as a maladaptation or protective mechanism, or rather strictly arising due to pathology. It is also unclear if Ub plays a role in this process, and thus far no autophagy receptors have been identified to participate in mammalian nucleophagy. Several autophagy-related proteins, including the selective autophagy receptor p62 and autophagic adaptor ALFY, have been documented to shuttle between the nucleus and cytoplasm [90,168,172]; however, there is no clear-cut evidence for their role in nucleophagy yet.

Interestingly, recent studies identified SUMOylation to play an important role in cellular activities including nucleophagy. DNA damage was shown to facilitate the accumulation of SUMO E2 ligase UBC9, and SUMOylation of lamin A/C [173]. This modification mediated the interaction between lamin A/C and LC3B, thus promoting nucleophagy. The knockdown of UBC9 prevented SUMOylation of lamin A/C and its interaction with LC3B, which attenuated nucleophagy and resulted in the degradation of nuclear components and leakage of nuclear DNA. SUMOylation has been previously implicated in the autophagic degradation of the polyglutamine (polyQ) protein ataxin-3, thus suggesting a role for SUMOylation in aggrephagy [19].

**Table 1:** Ubiquitin dependent autophagy receptors and their function.

Autophagy Receptor	Substrates	Function and Associated Pathologies	Refs
SQSTM1/p62	General autophagy Protein aggregates Mitochondria Pathogens Lipids Peroxisomes Lysosomes	The most universal autophagy receptor, p62, is involved in cellular stress response and the clearance of protein aggregates, defective organelles as well as invading pathogens. Defects in p62 are associated with Paget disease of bone, amyotrophic lateral sclerosis, and frontotemporal lobar degeneration	[58,59,80,159,174,175]
NBR1	Protein aggregates Mitochondria Peroxisomes	NBR1 is involved in aggrephagy and mitophagy, but is the main receptor for pexophagy	[60,61,62,130,176]
OPTN	General autophagy Protein aggregates Mitochondria Pathogens	OPTN acts as an autophagy receptor for several substrates. Its phosphorylation by TBK1 enhances its function thus facilitating the clearance of Salmonella; it acts as primary mitophagy receptor; mutations in OPTN were found to cause amyotrophic lateral sclerosis, and OPTN in present in protein inclusions found in several neurodegenerative diseases	[53,78,146]
NDP52	Mitochondria Pathogens	NDP52 interacts with LC3-C via noncanonical LIR motif and facilitates autophagosome maturation. It is the primary mitophagy receptor and collaborates with p62 for pathogen clearance	[62,158,177]
TAX1BP1	MitochondriaPathogens Lysosomes	Promotes autophagy flux in activated T cells, and is recruited to damaged lysosomes facilitating their elimination	[143,178]
TOLLIP	Protein aggregates	Facilitates degradation of protein aggregates such as huntingtin-derived polyQ proteins	[95]

## 15. Conclusions

Ubiquitin is a universal degradation signal used to tag proteins, organelles, and pathogens for disposal by the ubiquitin-proteasome and autophagy-lysosome systems. A large number of selective organelle-phagies are regulated, at least in part, by ubiquitination or other ubiquitin-like modifications. Ubiquitin acts as a beacon for the arrival of selective cargo receptors, and although the various selective types of autophagy rely on specific E3 ligases, of which there are upwards of 600, only a handful of Ub-dependent selective autophagy receptors have been identified thus far. Moreover, selective autophagy relies on common core autophagy machinery for the induction and formation of autophagosomes. This suggests common elements amongst the various types of selective autophagy which have likely evolved from bulk autophagy.

Despite the remarkable growth of knowledge in the field, spearheaded by talented “autophagians”, there is still much work to be done. The continuous identification of new selective types of autophagy must be followed up by a detailed characterization of the molecular mechanisms governing them. Likewise, novel cargo receptors involved in the various types of selective autophagy remain to be identified and characterized. Unraveling the molecular mechanisms that regulate the spatio-temporal ubiquitin signaling that controls selective autophagy, will be of great importance, to better understand its role in physiological and pathological processes.
